# Hemostasis Laboratory Diagnostics in Newborns

**DOI:** 10.3390/jcm14145068

**Published:** 2025-07-17

**Authors:** Chiara Gorio, Angelo Claudio Molinari, Tiziano Martini, Antonietta Ferretti, Giulia Albrici, Giulia Carracchia, Antonella Ierardi, Marzia Leotta, Nicola Portesi, Monica Sacco, Alessandra Strangio, Maria Elisa Mancuso, Rita Carlotta Santoro

**Affiliations:** 1 Pediatric Onco-Hematology Unit, Children’s Hospital, ASST Spedali Civili di Brescia, 25123 Brescia, Italy; chiaragorio@yahoo.it (C.G.); giulia.albrici92@gmail.com (G.A.); dottgiulia84@hotmail.it (G.C.); 2 Thrombosis and Hemostasis Unit, IRCCS Istituto Giannina Gaslini Children’s Hospital, 16147 Genoa, Italy; 3 Regional Reference Centre for Inherited Bleeding and Thrombotic Disorders, Transfusion Medicine—Città della Salute e della Scienza University Hospital, 10126 Turin, Italy; tizianom181@gmail.com; 4 Center for Hemorrhagic and Thrombotic Diseases, Foundation University Hospital “A. Gemelli” IRCCS, 00136 Rome, Italy; antonietta.ferrettii@gmail.com (A.F.); monicasacco.89@gmail.com (M.S.); 5 Hemostasis and Thrombosis Unit, Azienda Ospedaliero Universitaria Renato Dulbecco, 88100 Catanzaro, Italy; antonellaierardi86@gmail.com (A.I.); marzialeotta@virgilio.it (M.L.); alestr87@gmail.com (A.S.); ritacarlottasantoro@gmail.com (R.C.S.); 6 Clinical Chemistry Laboratory, Diagnostic Department, ASST Spedali Civili di Brescia, 25123 Brescia, Italy; nicola.portesi@asst-spedalicivilit.it; 7 Department of Transitional Medicine and Surgery, Catholic University of the Sacred Heart, 00153 Rome, Italy; 8 Center for Thrombosis and Hemorrhagic Diseases, IRCCS Humanitas Research Hospital, Rozzano, 20089 Milan, Italy; mariaelisa.mancuso@humanitas.it

**Keywords:** newborn, hemostasis, developmental hemostasis, bleeding disorders, coagulation tests, viscoelastic tests

## Abstract

The hemostatic system in the newborn is a complex entity, characterized by dynamism in its development; therefore, the correct measurement of its potential is challenging. In this narrative review, we analyzed the current knowledge of the “developmental hemostasis” of the newborn; we also studied the performance of routine coagulation tests in its evaluation, with considerations about the establishment of neonatal age-specific normal ranges and about the role of preanalytical variables, in particular, hematocrit (which could represent an important cause of error); we also focused on the increasing importance of viscoelastic coagulation tests, which are becoming increasingly widespread (especially in some settings such as intensive care unit) and are able to quickly provide information about the hemostatic function of the newborn, even if they lack adequate standardization in the neonatal period.

## 1. Hemostatic System Evolution—Trend of Procoagulants and Anticoagulants

Hemostasis in the newborn is a complex and dynamic system, which is reflected in the difficulty in interpreting the coagulation tests that are usually adopted; for this reason, it represents a challenge for both clinicians and laboratory workers. The aim of the present review is to examine the available evidence in this field and to try to make some suggestions regarding the correct performance and interpretation of the main coagulation tests.

### 1.1. Introduction: “Developmental Hemostasis”

Hemostasis is a complex phenomenon whose principal function is to mitigate the risk of excessive bleeding at sites of vascular injury. This process can be classified into primary (the adhesion, activation, and aggregation of platelets at a site of vessel injury), secondary (the activation of coagulation factors resulting in the formation of covalently cross-linked fibrin that stabilizes the platelet plug), and tertiary hemostasis (fibrinolysis, the activation of fibrinolytic pathway to dissolve the clot, restoring the physiological blood flow) [1]. The hemostatic system of the newborn greatly differentiates from those of both older children and adults: the principal characteristic of the hemostatic system in newborns is that it is dynamically and rapidly evolving over the first weeks and months of life. The term “developmental hemostasis” was coined in 1987 by Andrew, who demonstrated the differences in plasma levels and activity of coagulation proteins and the gradual increase in these from preterm to mature newborns [2,3]. This term is still used to define the peculiarity of the hemostatic system as it changes and matures from fetal life to childhood and adult life [4]. Table 1 summarizes the evolution of the hemostatic system components in the fetus and neonate.

### 1.2. Fetal Hemostasis

#### 1.2.1. Fetal Primary Hemostasis

The Von Willebrand factor (VWF) is expressed early in fetal life (4 weeks of gestation), reaching about 40% of adult levels at around 12–15 weeks of gestation; ultra-large multimers of VWF are found in fetal blood till 35 weeks of gestation, owing to decreased cleavage activity by the metalloproteinase ADAMTS-13. Therefore, fetal VWF prompts enhanced platelet adhesion to the sub-endothelium in high-shear-flow conditions [5]. Platelets can already be detected in fetal plasma at 11 weeks of gestation, and they reach the normal adult range at around 20 weeks of gestation [7]. The expression of surface receptors is already equivalent to adult platelets at 12–16 weeks of gestation, with the only exception being the epinephrine ones [7].

#### 1.2.2. Fetal Coagulation System

Coagulation proteins are independently synthesized by the fetus and do not cross the placenta [8]. Most of them are early expressed in embryonic and fetal development: factor VIII and fibrinogen can already be detected at 4–8 weeks of gestation, and they reach the normal adult range by midgestation, while factors VII, V, and XIII show progressive development during gestation [5,6]. The vitamin K-dependent proteins, factors II, VII, IX, and X, protein C, and protein S show decreased concentrations during fetal maturation (with a gradient for vitamin K across the placenta determining fetal levels as about 10% of maternal concentrations), ranging to from 10% to 20% of adult levels at midgestation [5,6]. Contact factors (factor XII, high-molecular-weight kininogen, prekallikrein) are present at low levels. Fetal fibrinogen shows a peculiar structure due to specific post-translational modifications: it is characterized by increased phosphorylation and sulfation, which lead to decreased clot strength [7].

#### 1.2.3. Fetal Fibrinolysis

Fibrinolytic activity can be detected in fetal plasma at 10–11 weeks of gestation [5]. In addition, plasminogen is present in a unique fetal form that contains increased concentrations of sialic acid, which confers a reduced tendency to activation [7]. Plasmin from fetal plasminogen has less enzymatic activity compared to adult plasmin [1].

### 1.3. Neonatal Hemostasis

#### 1.3.1. Neonatal Primary Hemostasis

Levels of VWF ultra-large and high-molecular-weight multimers, as well as VWF collagen-binding activity, are increased during the first 2 months of life and then gradually decrease to adult levels [9]. The maturation to typical adult VWF multimerics is similar in neonates and adults, with a reduced expression of platelet surface receptors (GPIIbIIIa, GPVI) as measured by flow cytometry and gene expression analysis. This could explain neonatal platelet hyporeactivity as compared to adult platelets [10]; however, through mass spectrometry, no significant differences in the expression level of major glycoproteins were observed in neonates [11]. It is noteworthy that the results of studies on neonatal platelet function can vary greatly according to the source of platelets: platelet count, mean platelet volume (MPV), and platelet surface glycoprotein expression (measured by flow cytometry) were found to be comparable between cord blood (CB) and peripheral blood (PB), while there were differences between CB and PB platelet activation (bound fibrinogen, CD63 and P-selectin) after agonist stimulation measured by flow cytometry and impedance platelet aggregation measured using ROTEM. Platelet aggregation was observed to be significantly higher in CB than PB [12]. The extent to which the technique of blood sampling varies from the two sources remains unclear, and further investigation is warranted [10]. However, these findings suggest that UC is a reliable alternative for basic parameters such as platelet count or GP expression, especially when minimal blood volume is a concern. However, using CB for evaluating functional platelet responses may misrepresent the in vivo hemostatic potential of neonatal platelets. The elevated aggregation seen in CB may be influenced by maternal blood components or vascular/endothelial factors not present in PB samples.

From a clinical standpoint, relying on CB for functional assessments could lead to inaccurate diagnoses or the misinterpretation of bleeding risk in neonates. For instance, enhanced aggregation in CB might mask underlying platelet dysfunction that is present in peripheral circulation.

For laboratory standardization, these discrepancies underscore the importance of clearly defining the blood source. Without standardization, comparisons across studies or between patients could be confounded, especially given the influence of sampling site on functional outcomes.

Thus, although CB can offer logistical advantages, PB remains the best source for assessing platelet function in term neonates due to its greater physiological relevance [12].

#### 1.3.2. Neonatal Coagulation

Postnatal levels of vitamin K-dependent factors (factors II, VII, IX, and X, protein C, and protein S) are significantly reduced compared to adult ones: factor IX completes its maturation at about 9–12 months (making the diagnosis of moderate and mild forms of Hemophilia B difficult) [5]. In the absence of postnatal vitamin K administration, a small number of neonates will show clinically significative bleeding related to the absence of vitamin K-dependent factors, with a very low incidence of life-threatening hemorrhages (intracranial, gastrointestinal) [5]. The levels of contact factors (factor XII, high-molecular-weight kininogen, prekallikrein) gradually increase, to approach adult levels by 6 months of life [8]. The plasma levels of fibrinogen, factor V, and factor XIII are already within the normal adult range at birth [8]; VWF and factor VIII can be normal or increased [2]. The levels of anticoagulants are reduced at birth compared to adults: antithrombin and protein C are about 50% of adult levels [1]; protein C is present in a fetal form (which is functionally active), increasing slowly to reach adult values by 13–14 years of age [1]. The protein S level is reduced at birth, but its activity is conserved, being present mainly in a free form because of the absence of C4b-binding protein [1]. Free TFPI levels are reduced (50–60% of adult values) [1].

#### 1.3.3. Neonatal Fibrinolysis

Neonates show an increased fibrinolytic activity: tPA has increased activity as compared to adults, although plasminogen levels are about 50% of adult levels at birth and rise to the normal adult range by 6 months of age [1].

### 1.4. Hemostasis of the Healthy Preterm Neonate

The World Health Organization (WHO) defines preterm neonates those babies born alive before the completion of the 37th week of gestational age (GA) [13]. The estimated rate of preterm birth is 4–16% across different countries. Several studies have analyzed the levels of coagulation factors in premature infants. Andrew et al. in 1988 studied 137 healthy preterm infants aged 30–36 weeks [3] and reported that vitamin K-dependent factors, protein C, protein S, antithrombin, and contact factors were <50% of adult levels, except for factor VII (67% of adult levels), factor VIII, and VWF, which were near or above adult values. More recently, Poralla et al. analyzed extremely preterm infants (from 23 to 27 weeks of GA) [14] and reported that factors VII and X rise with increasing GA, whereas fibrinogen and factors II, V, and VIII remain rather stable. Another study by Hochart et al. [15] reported that fibrinogen, factor II, and factor V rise with GA. Platelets from preterm neonates have reduced GPIIbIIIa activation, reduced fibrinogen binding and degranulation, resulting in decreased platelet function compared to platelets from term neonates [10]. Also in this setting, the platelet source (i.e., cord or peripheral blood) may impact the final results.

## 2. Preanalytical Variables: Role of Hematocrit

In neonates, high hematocrit (Hct) may be associated with conditions like dehydration, polycythemia, or certain physiological adaptations after birth (e.g., asphyxia). It is critical that we understand to what extent high hematocrit can influence laboratory results, particularly in coagulation studies, to avoid diagnostic errors. High hematocrit levels in neonates can have significant implications for coagulation testing, potentially leading to inaccurate or misleading results, particularly due to their impact on the balance between citrate anticoagulant and ionized calcium in the blood sample [16]. Hematocrit refers to the percentage of blood volume occupied by red blood cells (RBCs), and when it exceeds 55%, the reduced plasma volume in the sample leads to a lower concentration of ionized calcium. This, in turn, can impair clotting tests such as the prothrombin time (PT) and activated partial thromboplastin time (APTT), potentially causing falsely prolonged results. The altered anticoagulant-to-plasma ratio in such samples mimics the effect of underfilled tubes, where the anticoagulant disproportionately affects the plasma, leading to inaccurate test outcomes [17]. To mitigate this, guidelines from organizations such as the Clinical and Laboratory Standards Institute (CLSI) recommend adjusting the citrate-to-blood ratio in samples with hematocrit values above 55%. Specific formulas have been developed to guide this adjustment, ensuring accurate coagulation results even in patients with high hematocrit. The formula is as follows:

C = (1.85 × 10^−3^)(100 − Hct)(V)

where C is the volume of citrate in the tube, Hct is the patient’s hematocrit, V is the blood amount to be collected, and 1.85 × 10^−3^ is a constant [18]. However, this issue is less of a concern in cases of severe anemia, where the increased plasma volume allows sufficient calcium to remain available, thus preventing clotting interference [19]. Therefore, the proper adjustment of the anticoagulant is critical in neonates and others with high hematocrit to avoid diagnostic inaccuracies in coagulation testing; possible consequences of a misinterpretation of a falsely prolonged PT or APTT due to hematocrit interference include unnecessary second-level exams (e.g., clotting factor activity measurement), increased costs, more time taken, an increased risk of an iatrogenic anemia, the delaying of important (and, in some situations, lifesaving) invasive procedures such as central line placement, lumbar puncture, or surgeries, and unnecessary plasma transfusions (with the possibility of related adverse effects); these things can lead to a sense of anxiety and loss of trust among parents.

## 3. The Dynamic Hemostatic System of the Newborn and Its Effect on Laboratory Tests

The developmental hemostatic changes in the functional level of the coagulation proteins lead to several challenges for the clinician in correctly diagnosing a child with a coagulation disorder and in choosing and monitoring anticoagulant therapy [20,21,22]. Coagulation laboratory tests should be carefully interpreted due to the highly heterogeneous ranges seen between neonates compared with children and adult subjects. Andrew et al., for the first time, introduced reference ranges for healthy neonates over 30 years ago [2]. Since then, several authors have tried to define the reference standards in children, including the use of updated reagents and emerging technique with automated systems [23,24,25,26]. Besides the complexity in interpreting the results, there are also challenges in the preanalytical phase and analytical phase (e.g., difficulties in venipuncture, the absence of a free-flowing blood sample that can result in falsely prolonged PT and APTT) [27]. Furthermore, the newborn and infant population in these studies has been quite small, preventing definite conclusions from being drawn. The screening coagulation tests, APTT and PT, are usually prolonged compared with the adult normal range, even when reported as a ratio with normal plasma (which is usually adult plasma) [20,28]. Furthermore, the reference ranges for APTTs will differ with each different reagent and analyzer system, often significantly. Fetal reference ranges for coagulation parameters were studied for different gestational age groups, and the median test results were between 10% and 30% of adult values, depending on the parameter evaluated, in fetuses between 19 and 23 weeks of gestation and progressively increased to levels between 10% and 50% in fetuses between 30 and 38 weeks of gestation [28]. Unfortunately, the laboratory reference ranges must be interpreted with caution due to the significant laboratory variability of reagents and instruments used, as shown by Monagle in 2006 [29]. In 2012, The Scientific and Standardization Committee of the International Society on Thrombosis and Hemostasis (ISTH) published recommendations for children, specifying that hemostasis laboratories must use population-, reagent-, and analyzer-specific reference ranges [23]. The only truly valid reference range would be an age-matched range specific to the reagents and instruments used. It seems impossible to ask each laboratory to establish its own references intervals for all coagulation parameters in its own technical conditions [23]. These laboratory reference standards should be obtained only in specific, multicenter studies carried out using new reagent/analyzer combinations.

## 4. Sensitivity of Reagents to Absolute and Relative Factor Deficiencies

The hemostatic system is not fully developed until 3–6 months of age; therefore, differences between adults and infants are not always pathological and may be physiological [30]. The routine use of laboratory tests in neonates is often called into question for several reasons: firstly, reagents are not standardized for this age group; secondly, there are practical issues such as problems with blood sampling and the volume of blood required for a complete test; and, thirdly, there is a lack of information on how to interpret the results [31].

Different reference intervals for coagulation tests should be used for adult and pediatric patients to avoid misdiagnosis, which can have serious consequences for patients and their families [30]. The ISTH also recommends that each laboratory defines the age-dependent reference ranges under its own technical conditions [29]. A reference interval established using the same analyzer and reagent systems (after a validation process) should be used by laboratories that are unable to establish their own reference intervals ex novo. In this setting, the population variance should also be considered (including population-specific variance, reagent-specific variance, and analyzer-specific variance). If there is no reference interval value for the analyzer/reagent combination used in that laboratory, then great care should be taken in the interpretation of coagulation test results in children; therefore, each laboratory should establish its own reference interval for healthy populations in appropriate age groups [30]. Not all PT and APTT reagents are equally sensitive to reductions in coagulation factors; as shown by Toulon et al., APTT reagents show varying sensitivities to deficiencies of different coagulating factors. This is thought to be due to differences in the type and concentration of activator or phospholipids used in the reagent [32]. Moreover, an international standardization of APTT reagents is not available [33].

## 5. Viscoelastic Coagulation Test in Newborns

Viscoelastic coagulation tests (VCTs), such as Thromboelastography (TEG)/Rotational Thromboelastometry (ROTEM), are used as point-of-care devices that provide information on clot formation and lysis, allowing the entire hemostatic process to be monitored. According to the cellular model of coagulation, the complex process of hemostasis with the interactions between procoagulant factors, fibrinolytic proteins, cellular components, and platelets can be analyzed globally. The main advantage of VCTs compared to conventional tests is their ability to assess the patient’s hemostatic state numerically and graphically in real time. They also require a small blood sample, which is very useful in the neonatal setting [34]. Similarly to what happens for conventional coagulation tests, VCT parameters are also affected by Hct levels: a “hypocoagulable” profile, expressed as a prolonged CT (clotting time) and CFT (clot formation time) in INTEM/EXTEM assays and reduced A5 (clot strength at 5 min) in INTEM/FIBTEM assays, was observed in neonates with higher hematocrit levels [35,36]; on the other hand, the impact of low hematocrit in patients with anemia should be considered as a possible cause of “hypercoagulability” in ROTEM test results [37]. During fetal growth, there is an increased synthesis of procoagulant and anticoagulant factors, especially after 34 weeks of gestation, which is often not complete at the time of birth. PT and APTT are often altered due to vitamin K deficiency. However, these tests do not detect a concomitant deficiency of coagulation inhibitors. The use of viscoelastic tests may be useful in the assessment of global coagulation status in these patients [31]. The use of VCT in newborns has increased in recent years. However, it is limited by the lack of reference values for newborns. Differences between infants and adults have been reported in some studies. An observational study was conducted by Amelio and colleagues to identify non-coagulopathic term and late preterm infants admitted to a level III Neonatal Intensive Care Unit (NICU) with a VCM test in the first 72 h of life. The authors found that clotting time (CT) was significantly associated with PT but not APTT. They noted some differences with adults, especially a shorter CT and CFT (clotting time) and higher alpha A10, A20, and L130 than adults, as well as differences in Maximum Clot Firmness (MCF) and L145. They concluded that neonates take a shorter time than adults to reach a significant clot, with 25% of neonates having an alpha angle above the upper reference limit. However, compared with adults, there are no significant differences in the late parameters of clot strength and fibrinolysis (MCF and L145) [38]. The umbilical cord blood seems to be an inadequate sample for VCT, as demonstrated by Raffaelli et al. They found a procoagulant imbalance in the placental blood compared to the venous counterpart of the infant [39]. Sokou et al. compared 198 full-term and 84 preterm newborns to look for differences: there were no significant differences in ROTEM values between preterm and term infants, except for LI60; this could be due to there being lower levels of fibrinolysis inhibitors in preterm infants [40]. As with many other parameters, neonatal reference ranges are difficult to find. Oswald et al. presented age-specific reference values for ROTEM. They found that coagulation initiation and clot formation are directly related to age, whereas clot strength and fibrinolysis are similar across age groups. The ROTEM parameters of infants aged 0–3 months showed an accelerated induction of coagulation with clot firmness within the normal limits for adults [41]. A recent review by Manzoni et al. [42] confirms the importance of viscoelastic tests as valuable tools in identifying an acquired coagulopathy in high-risk neonates and allowing the prompt, targeted optimization of blood products and anticoagulation therapy; thromboelastography can optimize fresh-frozen plasma transfusion in surgical neonates [43]. In conclusion, viscoelastic tests have been used in the neonatal setting, especially in the NICU, and could help clinicians to understand the in vivo hemostatic conditions of the newborn. Nevertheless, currently, there are no specific neonatal guidelines and standardized reference values, and this limitation prevents this test from being used as the gold-standard method for the evaluation of neonatal coagulation [41]. From the point of view of the feasibility and cost-effectiveness of viscoelastic tests, the large-scale deployment of ROTEM/TEG in routine practice is now technically quite feasible—particularly with fully automated, cartridge-based platforms—yet still hinges on up-front investment, cold-chain reagent supply, and user training; some single-center trials and decision-analytic models have shown that viscoelastic-guided transfusion algorithms can reduce blood component use and shorten NICU stays, translating into net costs optimization [44]. Related to this aspect is the possible employment of TEG/ROTEM in neonatal units in low- and middle-income countries (LMICs), which face some broad hurdles. First of all, there are the costs, both of the purchase of the analyzers (they cost in the order of USD several thousand, which is prohibitive for many LMIC neonatal ICUs operating on limited budgets) and of the consumables (cuvettes or cartridges). Furthermore, supply chain fragility is as factor: reagents have short shelf lives and require cold-chain distribution. Then, there is the issue of infrastructure: reliable utilities (both systems demand continuous power and laboratory-grade bench space), cold-storage and calibration (reagents must be stored at 2–8 °C, and instruments require routine calibration and preventive maintenances, services that are often unavailable locally), and quality control programs (ROTEM and TEG must run daily QC checks to ensure accuracy). Finally, training and human resources are required, in particular for data interpretation: TEG/ROTEM outputs require the nuanced interpretation of results in the context of neonatal “developmental hemostasis” reference ranges (expertise that neonatologists and laboratory technicians rarely possess without targeted education) [45,46,47].

## 6. Conclusions

The evaluation of the real hemostatic competence of a newborn candidate in relation to an invasive or surgical procedure and the use of prolonged coagulation screening tests is a challenging and demanding situation for those who must manage such patients. Few hospitals have established normal ranges for coagulation tests for the neonatal period, and an expert in neonatal and pediatric hemostasis is not always available. Clotting factor measurements are not always available everywhere, while neonatal emergencies can occur in any hospital and at any time. Furthermore, hematocrit can constitute an important cause of preanalytical alteration. In addition, it is a current practice to administer thawed whole plasma to correct the apparent coagulopathy of the newborn before surgery; however, this strategy is not free from risks such as allergic reactions, infections, volume overload, incompatibility reactions, fever, and complications due to citrate, which is commonly contained in the bags. Global coagulation tests carried out with viscoelastic methods, which are increasingly widespread, especially in intensive care units and operating theaters, can help clinicians to quickly evaluate the hemostatic situation of neonates and infants, as well as providing information on platelet function even if, when carried out on citrated blood, these tests are prone to interference from the hematocrit. However, viscoelastic tests lack adequate standardization, especially in the neonatal age, and adequate studies are needed to demonstrate their ability to reveal mild coagulopathies, which, in the neonate, can be difficult to identify with conventional screening tests.

## Figures and Tables

**Table 1:** Hemostatic changes during fetal development and in the neonate [4,5,6].

Hemostasis Phase	Component	Time of Appearance in Fetal Period	Neonatal Period	Normalization	Effect on Neonatal Hemostasis
Primary hemostasis	Platelet	11 weeks; normal expression of surface receptors	Reduced activity	1 year	Enhanced primary hemostasis
	VWF	4 weeks; increased activity with increased ultra-large multimers	Increased activity with increased ultra-large and high-molecular-weight multimers	3 months	
Coagulation	FII, FVII, FIX, FX	10–11 weeks; 10–20% of adult levels at midge station	Decreased (40–60% of adult levels at birth)	9–12 months	Decreased coagulation activity
	FXI, FXII, PK, HMWK	10–11 weeks	Decreased	6 months	
	FVIII	4–8 weeks	Normal or increased	1 month	
	FV	10–11 weeks	Normal or slightly decreased	1 year	
	Fibrinogen	4–8 weeks	Normal or slightly decreased	1 year	
	Factor XIII	10–11 weeks	Normal	/	
	Antithrombin	10–11 weeks	Decreased (50% of adult levels at birth)	3 months	Decreased coagulation inhibition
	Protein C	10–11 weeks	Decreased (50% of adult levels at birth)	13–14 years	
	Protein S	10–11 weeks	Decreased (30–40% of adult levels at birth)	3 months	
Fibrinolysis	Plasminogen	10–11 weeks	Decreased (50% of adult levels at birth)	6 months	Increased fibrinolysis
	tPA	10–11 weeks	Increased	1 week	

## Data Availability

No new data were created or analyzed in this study. Data sharing is not applicable to this article.

## Abbreviations

The following abbreviations are used in this manuscript:

VWF	Von Willebrand Factor
WHO	World Health Organization
GA	Gestational Age
HCT	Hematocrit
PT	Prothrombin Time
APTT	partial thromboplastin time
RBCs	Red Blood Cells
PB	Peripheral Blood
CB	Umbilical Cord Blood
CLSI	Clinical and Laboratory Standards Institute
ISTH	International Society on Thrombosis and Hemostasis
VCT	Viscoelastic Coagulation Tests
TEG	Thromboelastography
ROTEM	Rotational Thromboelastometry
CT	Clotting Time
CFT	Clot Formation Time
NICU	Neonatal Intensive Care Unit

## References

[1] Saracco P., Rivers R.P.A. (2018). Pathophysiology of Coagulation and Deficiencies of Coagulation Factors in Newborn. Neonatology.

[2] Andrew M., Paes B., Milner R., Johnston M., Mitchell L., Tollefsen D.M., Powers P. (1987). Development of the human coagulation system in the full-term infant. Blood.

[3] Andrew M., Paes B., Milner R., Johnston M., Mitchell L., Tollefsen D.M., Castle V., Powers P. (1988). Development of the human coagulation system in the healthy premature infant. Blood.

[4] Toulon P. (2016). Developmental hemostasis: Laboratory and clinical implications. Int. J. Lab. Hematol..

[5] Manco-Johnson M.J. (2005). Development of hemostasis in the fetus. Thromb. Res..

[6] Manco-Johnson M.J. (2007). Development of hemostasis in the fetus and neonate. Thromb. Res..

[7] Warren B.B., Moyer G.C., Manco-Johnson M.J. (2023). Hemostasis in the Pregnant Woman, the Placenta, the Fetus, and the Newborn Infant. Semin. Thromb. Hemost..

[8] Monagle P., Massicotte P. (2011). Developmental hemostasis: Secondary hemostasis. Semin. Fetal. Neonatal. Med..

[9] Kuhle S., Male C., Mitchell L. (2003). Developmental Hemostasis: Pro- and Anticoagulant Systems during Childhood. Semin. Thromb. Hemost..

[10] Van Den Helm S., McCafferty C., Letunica N., Chau K.Y., Monagle P., Ignjatovic V. (2023). Platelet function in neonates and children. Thromb. Res..

[11] Stokhuijzen E., Koornneef J.M., Nota B., van den Eshof B.L., van Alphen F.P.J., van den Biggelaar M., van der Zwaan C., Kuijk C., Mertens K., Fijnvandraat K. (2017). Differences between Platelets Derived from Neonatal Cord Blood and Adult Peripheral Blood Assessed by Mass Spectrometry. J. Proteome. Res..

[12] Grevsen A.K., Hviid C.V.B., Hansen A.K., Hvas A.M. (2021). Platelet count and function in umbilical cord blood versus peripheral blood in term neonates. Platelets.

[13] WHO Recommendations for Care of the Preterm or Low-Birth-Weight Infant. https://www.who.int/publications/i/item/9789240058262.

[14] Poralla C., Traut C., Hertfelder H.J., Oldenburg J., Bartmann P., Heep A. (2012). The coagulation system of extremely preterm infants: Influence of perinatal risk factors on coagulation. J. Perinatol..

[15] Hochart A., Nuytten A., Pierache A., Bauters A., Rauch A., Wibaut B., Susen S., Goudemand J. (2019). Hemostatic profile of infants with spontaneous prematurity: Can we predict intraventricular hemorrhage development?. Ital. J. Pediatr..

[16] Kitchen S., Adcock D.M., Dauer R., Kristoffersen A.-H., Lippi G., Mackie I., Marlar R.A., Nair S. (2021). International Council for Standardisation in Haematology (ICSH) recommendations for collection of blood samples for coagulation testing. Int. J. Lab. Hematol..

[17] Marlar R.A., Potts R.M., Marlar A.A. (2006). Effect on routine and special coagulation testing values of citrate anticoagulant adjustment in patients with high hematocrit values. Am. J. Clin. Pathol..

[18] CLSI H21-A5—Collection, Transport, and Processing of Blood Specimens for Testing Plasma-Based Coagulation Assays and Molecular Hemostasis Assays; Approved Guideline-Fifth Edition. https://webstore.ansi.org/standards/clsi/clsih21a5?srsltid=AfmBOoqKconVXAchwBNfs7V5zdQM6_AyhicoI0u0L0KJ88tpb0J0mpYN.

[19] Siegel J.E., Swami V.K., Glenn P., Peterson P. (1998). Effect (or lack of it) of severe anemia on PT and APTT results. Am. J. Clin. Pathol..

[20] Jaffray J., Young G. (2013). Developmental hemostasis: Clinical implications from the fetus to the adolescent. Pediatr. Clin. North. Am..

[21] Ignjatovic V., Ilhan A., Monagle P. (2011). Evidence for age-related differences in human fibrinogen. Blood Coagul. Fibrinolysis.

[22] Lippi G., Franchini M., Montagnana M., Guidi G.C. (2007). Coagulation testing in pediatric patients: The young are not just miniature adults. Semin. Thromb. Hemost..

[23] Monagle P., Barnes C., Ignjatovic V., Furmedge J., Newall F., Chan A., De Rosa L., Hamilton S., Ragg P., Robinson S. (2006). Developmental haemostasis. Impact for clinical haemostasis laboratories. Thromb. Haemost..

[24] Toulon P., Berruyer M., Brionne-François M., Grand F., Lasne D., Telion C., Arcizet J., Giacomello R., De Pooter N. (2016). Age dependency for coagulation parameters in paediatric populations. Results of a multicentre study aimed at defining the age-specific reference ranges. Thromb. Haemost..

[25] Attard C., van der Straaten T., Karlaftis V., Monagle P., Ignjatovic V. (2013). Developmental hemostasis: Age- specific differences in the levels of hemostatic proteins. J. Thromb. Haemost..

[26] Appel I.M., Grimminck B., Geerts J., Stigter R., Cnossen M.H., Beishuizen A. (2012). Age dependency of coagulation parameters during childhood and puberty. J. Thromb. Haemost..

[27] Monagle P., Ignjatovic V., Savoia H. (2010). Hemostasis in neonates and children: Pitfalls and dilemmas. Blood Rev..

[28] Reverdiau-Moalic P., Delahousse B., Body G., Bardos P., Leroy J., Gruel Y. (1996). Evolution of blood coagulation activators and inhibitors in the healthy human fetus. Blood.

[29] Ignjatovic V., Kenet G., Monagle P. (2012). Perinatal and Paediatric Haemostasis Subcommittee of the Scientific and Standardization Committee of the International Society on Thrombosis and Haemostasis. Developmental hemostasis: Recommendations for laboratories reporting pediatric samples. J. Thromb. Haemost..

[30] Di Felice G., Vidali M., Parisi G., Pezzi S., Di Pede A., Deidda G., D’Agostini M., Carletti M., Ceccarelli S., Porzio O. (2022). Reference Intervals for Coagulation Parameters in Developmental Hemostasis from Infancy to Adolescence. Diagnostics.

[31] Sokou R., Parastatidou S., Konstantinidi A., Tsantes A.G., Iacovidou N., Piovani D., Bonovas S., Tsantes A.E. (2024). Contemporary tools for evaluation of hemostasis in neonates. Where are we and where are we headed?. Blood Rev..

[32] Toulon P., Eloit Y., Smahi M., Sigaud C., Jambou D., Fischer F., Appert-Flory A. (2016). In vitro sensitivity of different activated partial thromboplastin time reagents to mild clotting factor deficiencies. Int. J. Lab. Hematol..

[33] Marlar R.A., Strandberg K., Shima M., Adcock D.M. (2020). Clinical utility and impact of the use of the chromogenic vs one-stage factor activity assays in haemophilia A and B. Eur. J. Haematol..

[34] Wikkelsø A., Wetterslev J., Møller A.M., Afshari A. (2016). Thromboelastography (TEG) or thromboelastometry (ROTEM) to monitor haemostatic treatment versus usual care in adults or children with bleeding. Cochrane Database Syst. Rev..

[35] Theodoraki M., Sokou R., Valsami S., Iliodromiti Z., Pouliakis A., Parastatidou S., Karavana G., Ioakeimidis G., Georgiadou P., Iacovidou N. (2020). Reference Values of Thrombolastometry Parameters in Healthy Term Neonates. Children.

[36] Westbury S.K., Lee K., Reilly-Stitt C., Tulloh R., Mumford A.D. (2013). High haematocrit in cyanotic congenital heart disease affects how fibrinogen activity is determined by rotational thromboelastometry. Thromb Res..

[37] Özdemir Z.C., Düzenli Kar Y., Gündüz E., Turhan A.B., Bör Ö. (2018). Evaluation of hypercoagulability with rotational thromboelastometry in children with iron deficiency anemia. Hematology.

[38] Amelio G.S., Raffaeli G., Amodeo I., Gulden S., Cortesi V., Manzoni F., Pesenti N., Ghirardello S., Mosca F., Cavallaro G. (2022). Hemostatic Evaluation With Viscoelastic Coagulation Monitor: A Nicu Experience. Front. Pediatr..

[39] Raffaeli G., Tripodi A., Manzoni F., Scalambrino E., Pesenti N., Amodeo I., Cavallaro G., Villamor E., Peyvandi F., Mosca F. (2020). Is placental blood a reliable source for the evaluation of neonatal hemostasis at birth?. Transfusion.

[40] Sokou R., Foudoulaki-Paparizos L., Lytras T., Konstantinidi A., Theodoraki M., Lambadaridis I., Gounaris A., Valsami S., Politou M., Gialeraki A. (2017). Reference ranges of thromboelastometry in healthy full-term and pre-term neonates. Clin. Chem. Lab. Med..

[41] Oswald E., Stalzer B., Heitz E., Weiss M., Schmugge M., Strasak A., Innerhofer P., Haas T. (2010). Thromboelastometry (ROTEM^®^) in children: Age-related reference ranges and correlations with standard coagulation tests. Br. J. Anaesth..

[42] Manzoni F., Raymo L., Bronzoni V.C., Tomaselli A., Ghirardello S., Fumagalli M., Cavallaro G., Raffaeli G. (2025). The value of thromboelastography to neonatology. Semin. Fetal Neonatal Med..

[43] Raffaeli G., Pesenti N., Cavallaro G., Cortesi V., Manzoni F., Amelio G.S., Gulden S., Napolitano L., Macchini F., Mosca F. (2022). Optimizing fresh-frozen plasma transfusion in surgical neonates through thromboelastography: A quality improvement study. Eur. J. Pediatr..

[44] Whiting P., Al M., Westwood M., Ramos I.C., Ryder S., Armstrong N., Misso K., Ross J., Severens J., Kleijnen J. (2015). Viscoelastic point-of-care testing to assist with the diagnosis, management and monitoring of haemostasis: A systematic review and cost-effectiveness analysis. Health Technol. Assess. (Rockv.).

[45] Manzoni F., Raffaeli G., Cortesi V., Amelio G.S., Amodeo I., Gulden S., Cervellini G., Tomaselli A., Colombo M., Artoni A. (2023). Viscoelastic coagulation testing in Neonatal Intensive Care Units: Advantages and pitfalls in clinical practice. Blood Transfus..

[46] Katsaras G.Ν., Sokou R., Tsantes A.G., Piovani D., Bonovas S., Konstantinidi A., Ioakeimidis G., Parastatidou S., Gialamprinou D., Makrogianni A. (2021). The use of thromboelastography (TEG) and rotational thromboelastometry (ROTEM) in neonates: A systematic review. Eur. J. Pediatr..

[47] Cannata G., Mariotti Zani E., Argentiero A., Caminiti C., Perrone S., Esposito S. (2021). TEG^®^ and ROTEM^®^ Traces: Clinical Applications of Viscoelastic Coagulation Monitoring in Neonatal Intensive Care Unit. Diagnostics.

